# Choosing Not to Eat Meat: What Characterizes Independently Vegetarian Children?

**DOI:** 10.1111/desc.70292

**Published:** 2026-10-06

**Authors:** Matti Wilks, Alexa Sacchi, Beatrice Turner, Paul Bloom

**Affiliations:** ^1^ Department of Psychology University of Edinburgh Edinburgh UK; ^2^ Department of Psychology University of Toronto Toronto Canada; ^3^ Department of Psychology Yale University New Haven Connecticut USA

**Keywords:** altruism, compassion, cognition, developmental psychology, disgust, empathy, independent vegetarian, psychology, social dominance orientation, speciesism, vegetarian diets

## Abstract

A growing body of research has aimed to map the characteristics of extraordinary altruists—such as those who have donated a kidney to a stranger. However, this research has primarily focused on adults, and we know little about the development of extraordinary altruism. While children cannot donate their organs to strangers nor large portions of their income to charity, they can choose not to eat animals. In this study we examine *independent vegetarians* (i.e., children who become vegetarian while being raised in meat eating families) as a potential case‐study of extraordinarily altruistic motivation. In retrospective adult (Study 1) and developmental samples (Study 2) of American participants, we compare independently vegetarian children to family vegetarians and non‐vegetarians on measures of empathy/compassion, disgust sensitivity, cognitive reflection, fearful facial recognition, social dominance orientation, and speciesism. We find that both independent and family vegetarians were lower in speciesism and SDO, and higher in disgust sensitivity and empathy/compassion, with these differences emerging almost exclusively in the retrospective adult sample. Crucially, and contrary to our hypotheses, we find no differences between independent and family vegetarians, and discuss potential interpretations of this null result. These data offer new insight into a small body of literature about independent vegetarians and show that similar traits are associated with both family and independent vegetarianism.

## Summary

- This project represents one of the first explorations of a possible expression of extraordinarily altruistic motivation in childhood
- We examined differences between independent, family, and non‐vegetarian children on a range of personality and cognitive traits
- Surprisingly, independent vegetarians did not differ from family vegetarians on any measured personality or cognitive traits
- Family and independent vegetarians sometimes differed from non‐vegetarians on measures of speciesism, SDO, disgust sensitivity, and empathy/compassion

## 1 Introduction

In February 2025, Aly Coyle became the 100th person in Northern Ireland to donate a kidney to a stranger, saving the life of a 6‐year‐old boy living in England (Watson 2025). Aly is not alone—it is estimated that more than 1,000 altruistic (i.e., non‐directed) kidney donations have occurred in the United Kingdom and ∼4000 in the United States between 2001 and 2022 (Busari and Garba 2022). Why would these donors choose to help a person they do not know and will likely never meet?

Extraordinary altruism, also termed costly altruism, extreme altruism, or unusual altruism, refers to selfless acts directed to a distant other that significantly exceed normal expectations of helping and often involve risks or significant cost to the altruists themselves (e.g., Marsh et al. 2014; Oliner and Oliner 1988; Rhoads et al. 2023). This includes spontaneous altruistic acts, such as saving a stranger from drowning, as well as deliberate, calculated acts, such as donating a kidney to a stranger or donating substantial portions of one's salary to charity. When asked about their motivations, these altruists describe a range of influences, from religious and faith‐based commitments to explicitly articulated utilitarian moral frameworks emphasizing the maximization of overall welfare (MacFarquhar 2015)

A growing body of research has aimed to understand what distinguishes these extraordinary altruists from the rest of us. In a seminal study, Marsh et al. (2014) found that non‐directed kidney donors were uniquely sensitive to fearful facial expressions, a difference reflected in heightened amygdala responses to fear but not to other emotions. More recent studies have also found that altruistic kidney donors experience greater self‐other overlap in neural representations of pain and threat (Brethel‐Haurwitz et al. 2018), show reduced self‐other discounting (Vekaria et al. 2017), and differ from non‐altruist controls in their beliefs about the fundamental good of humanity (Amormino et al. 2022).

Research into extraordinary altruism has also expanded to incorporate other organ donors (e.g., liver donation; Rhoads et al. 2023), participants in SARS‐CoV‐2 human challenge trials (Rose et al. 2021), humanitarian aid workers (Rhoads et al. 2023), Carnegie Hero Medal recipients (Rand and Epstein 2014), those who have engaged in heroic acts of rescue (Rhoads et al. 2023), and those who pledged to donate large portions of their income to effective charities (Wilks et al. 2024). Echoing the results with non‐directed kidney donors, these groups also appear to differ in moral values (Amormino et al. 2022; Walker and Frimer 2007; Wilks et al. 2024) moral motivation (Rand and Epstein 2014), personality traits (Matsuba and Walker 2004), and a range of cognitive capacities (Wilks et al. 2024).

While we now know much more about what characterizes extraordinary altruism in adults, far less is known about its developmental origins. What might this altruistic motivation look like in childhood? Children cannot choose to donate their kidneys or direct large sums of money to charity. But they can choose not to eat animals. And some do, even when they are raised by families who eat meat—a choice referred to as *independent vegetarianism* (Hussar and Harris 2010). We see fundamental commonalities between this choice and other forms of extraordinary altruism.

First, research suggests that independently vegetarian children are particularly motivated by animal welfare when asked about their diet, even more so than vegetarians raised in vegetarian families (Hussar and Harris 2010). It's not that these children dislike the taste of meat, or think it's unhealthy—instead they typically think eating meat is morally wrong. Second, this choice can come at a personal cost. These children are giving up a food they may enjoy eating. Relatedly, there is evidence that vegetarians (Minson and Monin 2012), including childhood vegetarians (Loarte Castro 2023), experience discrimination for their choices. Finally, this decision is unusual—while many children express high levels of moral concern for animals (Caviola et al. 2025; Hussar and Harris 2018; McGuire et al. 2023, 2026; Paruzel‐Czachura et al. 2024; Wilks et al. 2021), very few make the decision to stop consuming them.

There is surprisingly little research explicitly examining vegetarianism in children. Most work focuses on nutritional outcomes for vegetarian children (Schürmann et al. 2017) and traits associated with vegetarianism in adulthood (see Rosenfeld 2018; Ruby 2012 for reviews). Moreover, while extensive research has examined children's moral development in general (see Bloom 2013; Brownell 2013; Hepach and Warneken 2018 for review), to our knowledge, only two studies have examined this case of what we see as extraordinarily altruistic motivation in childhood (i.e., independent vegetarianism). Hussar and Harris (2010, 2018) examined the beliefs and moral judgments of independently vegetarian children (6–12 years). The authors find that, compared to family and non‐vegetarian children, independent vegetarians express more concern for animal welfare when explaining their own diet choices, but that all three groups saw meat consumption as a personal choice (Hussar and Harris 2010). In another study, all three groups made similar moral judgments about harm to animals—viewing it as more morally wrong than the equivalent harm to a familiar person (e.g., a sibling; Hussar and Harris 2018). This research pioneered the study of independent vegetarianism in children and gives important insights into how these children think about meat consumption and harm to animals. However, it does not tell us about the characteristics of independent vegetarians—that is, what kind of traits might be associated with this decision. Echoing research with extraordinary altruism in adults (e.g., Rhoads et al. 2023), that is the focus of our project.

## 2 The Current Project

Across two studies, we examine the cognitive and personality traits that differentiate independently vegetarian children from family vegetarians and non‐vegetarians. In Study 1, we sampled a retrospective adult population—that is, adults who belonged to one of these three diet categories during childhood. In Study 2, we sampled children aged 8–11 years who currently adhere to these diets. We selected six cognitive and personality traits that will be tested in both samples and across all diet categories: empathy/compassion, disgust sensitivity, fear identification, cognitive reflection, social dominance orientation (SDO), and speciesism. We chose these traits based on the previous literature on extraordinary altruism, as well as a two‐stage consulting process. The first stage included surveying ∼10 experts in moral psychology and moral development about their views on which traits would be relevant.^1^ The second stage involved an online, anonymous, public survey to seek feedback on the same topic. We discuss the selected measures below.

### 2.1 Justification of Measures

#### 2.1.1 Cognitive Reflection

The cognitive reflection test (Frederick 2005) measures an individual's tendency to override an intuitive response and engage in reflection when answering questions. Cognitive reflection has been linked to prosocial motivations. For example, people higher in cognitive reflection are more likely to choose socially efficient distribution methods rather than those that improve their own relative shares (i.e., increasing overall funds rather than funds for themselves; Capraro et al. 2017). Moreover, past work has shown that similar traits, such as active open‐minded thinking and need for cognition, are higher in people who have taken the *Giving What We Can* 10% pledge to donate at least 10% of their income to effective charity (Wilks et al. 2024). As such, we predict that independently vegetarian children will be higher in cognitive reflection than family and non‐vegetarian children.

#### 2.1.2 Identification of Fearful Faces

Past work has shown that the ability to recognize fearful faces sets apart extraordinary altruists—both non‐directed kidney donors (Marsh et al. 2014) and *Giving What We Can* pledgers (Wilks et al. 2024)—from the general public. Moreover, accurate identification of fearful faces, but not other faces, is predictive of prosocial behavior in experimental tasks (Marsh et al. 2007). In line with this, we predict that children who are independently vegetarian will be more accurately able to identify fearful faces than family and non‐vegetarians.

#### 2.1.3 Disgust Sensitivity

Disgust sensitivity is an individual's sensitivity to stimuli that commonly evoke disgust responses (Haidt et al. 1994). Developmental research shows that disgust sensitivity appears to emerge by age five (Rottman 2014). Research with adults finds clear links between meat avoidance and disgust sensitivity (Becker and Lawrence 2021)—though the direction of this effect remains unclear (Becker et al. 2022; Buttlar and Walther 2022). However, to our knowledge, no research has explicitly linked vegetarian diet and disgust in childhood. Nonetheless, we predict that independently vegetarian children will be more disgust sensitive than family and non‐vegetarians.

#### 2.1.4 Empathy/Compassion

Our tendency to feel compassion and empathy for others is frequently related to prosocial behavior (Batson et al. 2007; Leiberg et al. 2011), though this relationship is not always consistent (Jordan et al. 2016; Preylo and Arikawa 2008; Wilks et al. 2024). However, research with children shows that they form strong connections with animals (Hawkins and Williams 2017), and one study found that children with pets were more empathetic than those without (Vidović et al. 1999). Moreover, independently vegetarian children cite moral reasons for not eating meat, and children generally report that it is wrong to harm animals because they are vulnerable (Hussar and Harris 2018). As such, we predict that independent vegetarians will be higher in empathy and compassion than family and non‐vegetarians.

#### 2.1.5 Social Dominance Orientation

SDO is a measure of an individual's preference for hierarchy within a social system (Pratto et al. 1994). Many studies with adults have linked veganism to lower rates of SDO (Allen et al. 2000; Braunsberger et al. 2021; Hodson and Earle 2018; Singer et al. 2015). Moreover, higher levels of SDO are related to other hierarchical worldviews such as racism, homophobia and, crucially, speciesism (Caviola et al. 2019). Finally, prior work shows that *Giving What We Can* pledgers are lower in SDO than the general population (Wilks et al. 2024). While research on SDO in children is limited, existing studies find positive links between SDO and empathy (Cadamuro et al. 2022). As such, we predict that independently vegetarian children will be lower in SDO than family or non‐vegetarians.

#### 2.1.6 Speciesism

Speciesism is the tendency to assign moral worth based on species category (e.g., human), particularly prioritize humans over non‐human animals. Speciesism has been linked to a range of hierarchical worldviews, such as SDO and homophobia in adults (Caviola et al. 2019). Research also shows that children are generally less speciesist than adults (Henseler Kozachenko and Piazza 2021; Marshall et al. 2025; McGuire et al. 2023; Paruzel‐Czachura et al. 2024; Wilks et al. 2021). In line with this, we predict that independently vegetarian children will be lower in speciesism than family and non‐vegetarians.

### 2.2 Hypothesis Summary

We predict that independent vegetarians will differ from family and non‐vegetarians on all measures detailed above in both the retrospective adult and child samples. Specifically, independent vegetarians will score higher on measures of cognitive reflection, empathy/compassion, disgust sensitivity, be better at identifying fearful faces, and lower in speciesism and SDO.

Both studies were preregistered on the Open Science Framework: https://osf.io/r4uwm. All materials, data, and code are also available on the Open Science Framework: https://osf.io/xb2s9/. Ethical approval was granted by [redacted] and [redacted].

## 3 Study 1 (Adults)

### 3.1 Method

#### 3.1.1 Participants

We recruited adult participants from the United States via social media and snowball recruitment, targeting general pages and vegetarian‐oriented communities (e.g., Facebook, Twitter/X, Reddit). A power analysis revealed that to achieve 0.95 power and detect a medium effect size (*f* = 0.25) with an alpha of 0.05, a sample of *N* = 252 (84 per cell) was recommended (G*Power; Faul et al. 2007). A total of 681 adult participants accessed the survey. Two hundred and six were excluded for not completing the survey in full (stopping between consent and final questions), and 54 were excluded for completing most of the survey, but not specifying their diet information. Another 36 participants were removed from the survey for not meeting residency criteria (i.e., the United States).

This left a final sample of 385 participants (*M*_Age =_ 32.2 years, Age Range 18–73, SD = 10.9). Participants were grouped by their retrospective diet choices independent vegetarians (*N* = 111), family vegetarians (*N* = 97), and non‐vegetarians (*N* = 177; see dietary criteria below).

Our sample was comprised of more female (88%) than male (12%) participants in the independent and family vegetarian groups, which is reflective of higher rates of female vegetarianism found in the general population (e.g., Pfeiler and Egloff 2018; Rosenfeld 2018). The majority of participants were white (82.1%). The rest of the sample identified as multi‐racial (7.0%), other (2.9%), Indian (2.9%), Hispanic (2.3%), Asian (1.8%), Black (0.8%), and not reported (0.3%). As is typical of online samples, our sample skewed more non‐religious (83.8%) and more liberal than the typical population, with an average score of 7.4 on a Likert scale from 1 (very conservative) to 9 (very liberal).

#### 3.1.2 Dietary Criteria

We defined independent vegetarians as adults who were raised in non‐vegetarian families (i.e., no immediate family members identified as vegetarian) but who had decided to adopt a vegetarian diet before 10 years of age, and had maintained that diet for at least 3 months.^2^ We chose this to ensure that the sample was comparable to the developmental sample in Study 2 (i.e., where participants were 8–11 years of age). We defined family vegetarians as adults who, as children, ate a vegetarian diet along with at least one member of their immediate family. We defined non‐vegetarians as those who, as children, ate meat along with their immediate family. We specified that vegetarian refers to not consuming animal flesh, including meat, fish and seafood, but not other animal products (e.g., milk, eggs). We provided these criteria to participants and allowed them to self‐identify.

#### 3.1.3 Procedure and Materials

Participants completed the survey independently, online via the Qualtrics survey platform. After providing consent, participants completed the six cognitive and personality measures in a randomized order (see Table 1; the full versions of all scales and instructions used are available on the Open Science Framework). Participants then provided basic demographics (age, sex, ethnicity, political orientation, and religiosity). Subsequently, they responded to a series of questions about their diet, including their current diet, childhood diet category (retrospective or current), as either independent, family, or non‐vegetarian.

**TABLE 1:** Operationalization of measures used.

	Cronbach's alpha		
Measure	Study 1 (Adults)	Study 2 (Children)	Operationalization
Cognitive reflection (Thomson and Oppenheimer 2016)	*α* = 0.54	*α* = 0.46	Participants responded to four questions that aimed to capture either intuitive or reflective thinking. Responses were scored as either correct or incorrect. For example, *“If you're running a race and you pass the person in second place, what place are you in?”*. (Correct: *second place*; Incorrect: f*irst place*).
Identification of fearful faces (Adapted from Marsh et al. 2007)	*α* = 0.38	*α* = 0.32	This was measured as part of a larger emotion recognition task. Participants saw 16 forward facing images from the Radboud Faces Database (Langner et al. 2010), which comprised four images of four emotions: happy, angry, disgusted, and scared. Participants were asked to identify which of the four emotions was displayed. Responses were scored as correct or incorrect.
Disgust sensitivity (Olatunji et al. 2007)	*α* = 0.81	*α* = 0.59	The disgust measure includes two sections. Responses were aggregated across both sections. In section one, participants were asked to agree with 14 statements on a scale of *0 = Strongly disagree (very untrue about me) to 4 = Strongly agree (very true about me)*. The questions involved physical disgust, for example, *“Even if I was hungry, I would not drink a bowl of my favorite soup if it had been stirred with a used but thoroughly washed flyswatter.”* In the section two, participants rated their disgust to 13 scenarios on a 5‐point Likert scale *0 (Not at all disgusting)* to *4 (Extremely disgusting)*. These questions also involved physical disgust, including questions about food and bodily contamination, death, and sex. For example, *“You take a sip of soda and realize that you drank from the glass that an acquaintance of yours had been drinking from.”*
Empathy and compassion (Jordan et al. 2016)	*α* = 0.87	*α* = 0.84	This measure comprised four of the six subscales in the empathy and compassion scale (perspective taking, concern, empathy, behavioral contagion. Participants were asked to report how well 28 statements described them on a 5‐point Likert scale from *0 (Does not describe me at all)* to *4 (Describes me very well)*. Responses were aggregated across both sections. For example, *“I sometimes try to understand my friends better by imagining how things look from their perspective.”*
Social Dominance Orientation (SDO) (Ho et al. 2015)	*α* = 0.82	*α* = 0.60	Participants were asked to respond to 8 items, indicating how much they favor or oppose ideas on a 7‐point Likert scale *1 (strongly oppose)* to *7 (strongly favor)*. For example, *“An ideal society requires some groups to be on top and others to be on the bottom.”*
Speciesism (Caviola et al. 2019)	*α* = 0.84	*α* = 0.69	Participants reported how much they agreed with 6 statements on a 7‐point Likert scale ranging from *1 (Strongly Disagree)* to *7 (Strongly Agree)*. For example, *“Chimpanzees should have basic legal rights, like a right not to be hurt.”*

*Note*: Cronbach's alpha was low for the identification of fearful faces for Study 1 (*α* = 0.38) and Study 2 (*α* = 0.32). This is likely due to low variance in the responses on this scale, with a high proportion of participants answering correctly on all four items.

For both family and independently vegetarian participants, we asked at what age their diet choice began, and the motivation for their diet choice. We also asked whether anyone in their social network was vegetarian (immediate family, extended family, and friends). For participants who were vegetarians as children but later chose to eat meat, we asked about the motivation for their dietary change. At the completion of the study, participants were thanked and debriefed.

#### 3.1.4 Data Coding

All scales were coded in line with preregistered instructions which are provided in full on the Open Science Framework. Trained research assistants manually coded responses to the cognitive reflection test. The correct answer was coded as 1, and the incorrect answer was coded as 0. A second coder coded 25% of responses. Cronbach's alpha revealed that interrater reliability was good (*α* = 0.78).

## 4 Results and Discussion

### 4.1 Preregistered Confirmatory Analyses

To test our preregistered hypotheses, we conducted six ANCOVAs—for each of the six outcome variables (Table 2). In each model, we included religion, sex, and diet category as fixed factors, and political orientation as a covariate. For four of the outcome variables, we found no omnibus differences: for CRT (*p* = 0.766), identification of fearful faces (*p* = 0.695), disgust sensitivity (*p* = 0.631) nor SDO (*p* = 0.165). However, the ANCOVAs showed a significant difference for speciesism, *F* (2, 369) = 23.89, *p* < 0.001, *ηp* ^2^ = 0.12. A Tukey HSD follow up test revealed that non‐vegetarians (*M* = 2.19, SD = 1.31) were significantly more speciesist than independent vegetarians (*M* = 1.29, SD = 0.69) and family vegetarians (*M* = 1.51, SD = 0.89; both *p*s < 0.001). However, family vegetarians did not significantly differ from independent vegetarians, *p* = 0.696. Additionally, the omnibus ANCOVAs showed a significant difference for empathy/compassion, *F* (2, 369) = 3.50, *p* = 0.031, *ηp* ^2^ = 0.02. A Tukey HSD follow up test revealed that independent vegetarians (*M* = 77.36, SD = 14.75), were significantly higher in empathy/compassion than non‐vegetarians (*M* = 71.03, SD = 15.30; *p* = 0.023). Family vegetarians (*M* = 73.76, SD = 14.87) were not significantly different from either independent or non‐vegetarians (*ps* ≥ 0.329).

**TABLE 2:** Standard deviations and means from the six ANCOVAs, comparing outcomes for the six metrics of personality and cognition across the three dietary groups.

					Family vegetarians		Independent vegetarians		Non‐vegetarians	
	*F*	df1	df2	*p*	Mean	SD	Mean	SD	Mean	SD
Cognitive reflection (0–4)	0.27	2	369	0.766	2.24	1.04	2.16	1.26	2.29	1.08
Identifying fearful faces (0–4)	0.36	2	369	0.695	3.78	0.60	3.83	0.48	3.81	0.46
Disgust sensitivity (0–108)	0.46	2	369	0.631	56.31	13.63	57.23	12.98	54.92	14.42
Empathy/compassion (0–112)	3.50	2	369	**0.031**	73.76	14.87	77.36	14.75	71.03	15.30
SDO (1–7)	1.81	2	369	0.165	1.65	0.74	1.69	0.90	1.90	1.03
Speciesism (1–7)	23.89	2	369	**<0.001**	1.51	0.89	1.29	0.69	2.19	1.31

*Note*: Bolding indicates a significant p value.

### 4.2 Preregistered Exploratory Analyses

In line with our preregistration, we conducted a multinomial logistic regression including all six personality/cognitive scales, political orientation, and religiosity as predictors and childhood diet status (independent compared to family and non‐vegetarian) as the outcome measure. We also included sex as a predictor, given the high rates of vegetarian women in the general population (Pfeiler and Egloff 2018; Rosenfeld 2018). All continuous variables were mean‐centered.

Overall, the model provided good fit for the data, predicting diet category over and above the intercept only model, *χ^2^* (18) = 71.92, *p* < 0.001. Pseudo *R* ^2^ values ranged from 0.062 (Cox & Snell) to 0.090 (McFadden). With all six cognitive and personality measures, religiosity, political orientation, and sex included in the model, only speciesism and sex predicted diet category. A one‐unit increase in speciesism was associated with 2.37 times greater odds of being non‐vegetarian as compared to independent vegetarian (*B* = 0.86, 95% CI [0.53–1.19], *p* < 0.001). There were no differences between independent and family vegetarians for speciesism (*B* = 0.28, 95% CI [−0.09–0.66], *p* = 0.142). Sex was also shown to predict diet category such that males had 2.77 times greater odds of being non‐vegetarian compared to independent vegetarian, (*B* = 1.02, 95% CI [0.05–1.99], *p* = 0.039). The same went for family vegetarians, such that males were 3.69 times more likely to be family vegetarians compared to independent vegetarians than females, (*B* = 1.31, 95% CI [0.29–2.32], *p* = 0.011).

In line with our preregistration, we also report participants’ motivation for becoming independent or family vegetarians, as well as their reason for giving up vegetarianism, if applicable. A trained research assistant coded the responses for motivation to become vegetarian and motivation for no longer being vegetarian, and a second coder coded 100% of responses. Cronbach's alpha revealed that interrater reliability was good for the motivation to become vegetarian (*α* = 0.88) and the motivation for no longer being vegetarian (*α* = 0.82). See Table 3 for the breakdown of motivations for engaging in or giving up vegetarianism.

**TABLE 3:** Motivation for becoming vegetarian and giving up vegetarianism as a function of vegetarian type (family, independent).

	Motivation to become vegetarian		Motivation to give up vegetarianism	
	Family	Independent	Family	Independent
Health	2	4	2	0
Environment	0	2	0	0
Social	62	0	0	2
Animal welfare	27	92	0	0
Convenience	3	12	2	1
Other	3	1	0	0
Total	97	111	4	3

We preregistered that we would conduct chi‐square comparisons to see whether motivation varied as a function of vegetarian category (family vs. independent) if we had more than 20 participants per cell. Though most motivation categories did not meet this criterion, animal welfare had greater than 20 participants for family and independent vegetarians. Given the theoretical relevance of morally motivated vegetarianism to this paper, we opted to compare the frequencies of those who cited animal welfare as their primary motivation (*N* = 119), and all other vegetarian participants (*N* = 89) for family vegetarians and independent vegetarians. We found that independent vegetarians were significantly more likely to cite animal welfare motivations than family vegetarians, *χ* ^2^(1, *N* = 208) = 64.08, *p* < 0.001, (Fisher's exact *p* < 0.001), OR = 0.080, 95% CI [0.04–0.16].

We also noted potential differences between independent and family vegetarians in two of the other motivation categories (convenience and social factors). We opted to use chi‐square comparisons with Fisher's exact tests to account for the low number of participants in many cells. The results revealed that family vegetarians were significantly more likely to cite social factors than independent vegetarians, *χ* ^2^(1, *N* = 208) = 101.08, *p* < 0.001, (Fisher's exact *p* < 0.001) OR = 392.61, 95% CI [23.68–6510.64] (Haldane‐Anscombe correction applied due to a zero cell), while independent vegetarians were more likely to cite convenience than family vegetarians, *χ* ^2^(1, *N* = 208) = 4.61, *p* = 0.032, (Fisher's exact *p* = 0.035) OR = 0.26, 95% CI [0.07–0.96].

### 4.3 Exploratory Dietary Motivation

When making the case that independent vegetarianism could represent a form of extraordinary altruism, we cited their moral motivation as a central argument. In line with this, we opted to examine whether vegetarians who chose animal welfare as their primary motivator (moral vegetarians; *N* = 119) differ from all other participants (*N* = 266). This comprised vegetarians from both the independent (*N* = 92) and family (*N* = 27) categories. A series of Bonferroni corrected *t*‐tests (*α* = 0.008) revealed differences in empathy/compassion, SDO, and speciesism between the two groups (See Table 4). Morally motivated vegetarians scored higher in empathy/compassion and lower in both SDO and speciesism. There were no differences between moral vegetarians and all other participants for disgust sensitivity, identifying fearful faces, or cognitive reflection (*ps* > 0.287).

**TABLE 4:** Bonferroni‐corrected *t*‐tests were used to compare morally motivated vegetarians with all other participants for the six cognitive/personality measures.

					Moral vegetarians		Other		
	*t*	*p*	95% CI		Mean	SD	Mean	SD	Effect size
Cognitive reflection test	−0.63	0.528	−0.32	0.17	2.18	1.20	2.26	1.09	−0.07
Identifying fearful faces	1.07	0.287	−0.05	0.17	3.85	0.50	3.79	0.51	0.12
Disgust sensitivity	0.56	0.573	−2.14	3.86	56.53	13.25	55.67	14.08	0.06
Empathy/compassion	2.84	**0.005**	1.45	7.99	76.81	14.82	72.09	15.21	0.31
SDO	−2.99	**0.003**	−0.46	−0.09	1.58	0.77	1.86	0.98	−0.31
Speciesism	−8.31	**<0.001**	−0.97	−0.60	1.22	0.62	2.00	1.23	−0.81

*Note*: Levene's test for equality of variances was significant for SDO and speciesism, thus we report the statistics for equal variances not assumed using Welch's *t*‐test. Bolding indicates a significant p value.

### 4.4 Reanalysis with Consistent Diet

While examining the data, we noted that there was a high proportion of participants in the non‐vegetarian category who had chosen to become vegetarians after 11 years of age (*N* = 101/177, leaving *N* = 76). That is, we classified them as non‐vegetarian children, but they adhered to a vegetarian diet as adults at the time of participation. This is likely because our recruitment methods targeted vegetarian‐aligned communities on social media. It is possible that participants who became vegetarian after age 11 may respond differently from those who have been consistently non‐vegetarian since early childhood.

In light of this, we opted to re‐examine the main comparisons again with these vegetarian participants removed from the sample. These exclusions comprised of 101 participants who were non‐vegetarian in childhood but identified later in life as vegetarian, in addition to seven participants who abandoned their childhood vegetarian diet in adulthood. This left us with a final sample of 277 (*N* = 76 non‐vegetarians; *N* = 108 family vegetarians, *N* = 93 independent vegetarians) for the exploratory analysis.

We first re‐ran the six ANCOVAs. As per the full sample, significant differences were again revealed for empathy/compassion (*p* < 0.001) and speciesism (*p* < 0.001), but not for cognitive reflection (*p* = 0.955) nor accuracy of identifying fearful faces (*p* = 0.437). However, the pattern of results changed, revealing differences for SDO, *F*(2, 264) = 3.69, *p* = 0.026, *ηp* ^2^ = 0.03 and disgust *F*(2, 264) = 4.42, *p* = 0.013, *ηp* ^2^ = 0.03. Tukey HSD follow up tests revealed that non‐vegetarians (*M* = 2.20, SD = 1.11) were significantly higher in social dominance than family vegetarians (*M* = 1.62, SD = 0.73), *p* = 0.021, though independent vegetarians (*M* = 1.69, SD = 0.88) were not significantly different from family (*p* = 0.645) or non‐vegetarians (*p* = 0.146). Tukey HSD follow up tests revealed that non‐vegetarians (*M* = 50.76, SD = 13.65) were significantly lower in disgust than family (*M* = 56.51, SD *=* 13.63), *p* = 0.047, and independent (*M* = 57.52, SD = 12.95) vegetarians, *p* = 0.013, who were not significantly different from each other (*p* = 0.856).

We also re‐ran the multinominal logistic regression, again finding that the model provides a good fit for the data, *χ* ^2^ (18) = 140.72, *p* < 0.001. Pseudo *R* ^2^ values ranged from 0.16 (Cox & Snell) to 0.24 (McFadden). Notably this model appears to provide better fit than the previous model (which included the vegetarians in the non‐vegetarian category). Consistent with the full sample, speciesism (*p* < 0.001) and sex (*p* = 0.007) was again significant predictors of being independent vegetarian compared to non‐vegetarian, while only sex was a significant predictor of being family vegetarian compared to independent vegetarian (speciesism *p* = 0.127; sex *p* = 0.015). However, we now also find that empathy/compassion is a significant predictor of diet category where a one‐unit increase in empathy/compassion was associated with an approximately 3% decrease (odds ratio = 0.97) in the likelihood of being non‐vegetarian as compared to independent vegetarian, (*B = −*0.03, 95% CI [−0.06 to −0.005], *p* = 0.021). Empathy/compassion did not uniquely predict diet between independent and family vegetarians, *p* = 0.131). All other personality and cognitive traits or demographics remained non‐significant.

Finally, we re‐examined the *t*‐tests between moral (childhood) vegetarians and all other participants, again employing Bonferroni corrections to account for multiple comparisons (*p* = 0.008). The same pattern of results emerged, where moral vegetarians were higher in empathy/compassion and lower in speciesism and SDO (*p*s < 0.001), while we found no differences for disgust sensitivity, identifying fearful faces or cognitive reflection (*p*s > 0.102).

In sum, removing the vegetarian adults from the non‐vegetarian sample resulted in two key changes. First, in the linear regression, empathy/compassion uniquely predicted the diet category when distinguishing between independent and non‐vegetarians (but not family vegetarians), reflecting the pattern of results found in the ANOVA with the full sample. Second, in the ANOVAs, non‐vegetarians were found to score higher on SDO and disgust than family, but not independent, vegetarians. Notably, these results did not persist in the regression model. No changes were found comparing morally motivated vegetarians to the rest of the sample.

## 5 Discussion

In Study 1, we find no differences between family and independent vegetarians, but we do find a number of differences between vegetarians and to non‐vegetarians. With all participants included and controlling for demographics we found that independent and family vegetarians were lower in speciesism than non‐vegetarians, and that independent (but not family) vegetarians were higher in empathy/compassion, while no other traits were predictive. In a full regression model including all traits, speciesism remained a significant predictor, whereas empathy/compassion did not. Here too we found that sex was a predictor, with females more likely to be vegetarian than males. This likely reflects vegetarianism being more prevalent in females in the general population (Pfeiler and Egloff 2018; Rosenfeld 2018).

However, as we had a high number of people in the non‐vegetarian category who were now vegetarian (i.e., they had become vegetarian as adults), we opted to conduct another analysis with these participants removed (*N* = 101). Empathy/compassion remained predictive, and we also found that both independent and family vegetarians were lower in SDO and higher in disgust than non‐vegetarians when examined separately (i.e., ANOVAs). However, the latter findings did not persist when other variables and demographics were included our models.

We also examined the role of dietary motivation, with independent vegetarians reporting higher rates of animal welfare motivation than family vegetarians, consistent with past research (Hussar and Harris 2010). Independent vegetarians were also more likely to cite convenience and less likely to cite social factors than family vegetarians. Finally, in exploratory analyses, we revealed that morally motivated vegetarians were higher in empathy/compassion and lower in both SDO and speciesism, and that this persisted regardless of whether we included or excluded current vegetarians from the retrospective non‐vegetarian category.

Here, we provide the first insight into the characteristics of people who had vegetarian diets in childhood. However, there are limitations on what can be inferred from retrospective samples. To make stronger claims about independent vegetarianism in childhood, we also directly examined these traits in a sample of independent, family, and non‐vegetarian children.

## 6 Study 2 (Children)

In Study 1, we found that independent vegetarians differed from non‐vegetarians on speciesism, empathy/compassion, SDO, and disgust. They did not differ from family vegetarians on any measure. In the current study, we undertake the same exploration with a sample of children who adhered to these diet categories at the time of data collection.

### 6.1 Method

#### 6.1.1 Participants

We initially aimed to recruit 252 participants, in line with the preregistration. However, we were unable to recruit a sufficient sample of independently vegetarian children and, despite extensive advertising, opted to stop recruitment after multiple years of limited success. We ultimately recruited a total of 245 children aged 8–11 years old living in the United States. Participant's parents were recruited through advertisements on social media or from a database of parents who had previously agreed to participate in research. Study sessions lasted approximately 20‐min and all participants received a $10 Amazon gift card voucher as compensation.

Nine children were excluded for reasons such as parental interference (*N* = 3), voluntary withdrawal midway through the survey (*N* = 1), environmental distractions (*N* = 2), or failure to meet age or country of residence criteria, which was unknown until after the study's completion (*N* = 3). This left a final sample of 236 children (*M*_Age_ = 9.39 years, Age Range = 8–11 years, SD = 1.03). Unlike the adult sample, there was an even split among sex (female = 53%; male = 47%), contrasting with higher rates of vegetarianism among females in the general population (Pfeiler and Egloff 2018; Rosenfeld 2018).

Similar to the adult sample, children were classified into one of three diet categories: independent vegetarians (*N* = 64), family vegetarians (*N* = 88), and non‐vegetarians (*N* = 84). Again, we required that children have been engaging in a vegetarian diet for at least 3 months. Due to difficulty with recruitment, we broadened our criteria to include those who identified as pescatarian, vegetarian, vegan, or had mostly a vegetarian diet (i.e., only rarely ate meat). Additionally, we included independent vegetarian children who were the first in their family to choose a vegetarian diet, as we found through discussions with participants that some children's dietary choices were the catalyst for other family members to become vegetarian. We then cross‐referenced participants’ identification with their self‐reported diet (e.g., if they identified as an independent vegetarian, but also reported consuming meat regularly) and reasons for going vegetarian, adjusting their dietary categorization where necessary. For instance, if someone self‐reported themselves as an “independent vegetarian,” but later on it was revealed they became vegetarian because of a sibling they would then be reclassified as a “family vegetarian.” This occurred for 14 children.

Parents were asked to report their child's demographic information. The majority of participants were white (75.8%), with the rest of the sample indicating their race as multi‐racial (10.6%) Asian (4.2%), Black (2.5%), Hispanic (2.1%), other (2.1%). Indian (1.3%), and not reported (1.3%). The child sample was more religious than the adult sample, with 41.5% of the children's parents identifying as religious, 56.4% as non‐religious, and 2.1% not reporting religiosity.

#### 6.1.2 Procedure and Materials

Child participants met with trained experimenters who completed the study online via Zoom. Experimenters shared their screens so that participants could see the stimuli and read each question out loud. Children then answered verbally, and the experimenter indicated the child's answer on the survey.

Children completed the same six measures as adults in Study 1. However, minor changes were made to the SDO, disgust sensitivity, speciesism and empathy/compassion scales to ensure that they were child‐friendly (e.g., removing references to sex or human death and rewording to be more generally child‐friendly). All changes are documented on the Open Science Framework.

Parents completed demographic questions about their child, including a question confirming their child's diet. In addition, parents also completed a short survey about their parenting style. The survey was a subset of five items from the parenting practices questionnaire (Robinson et al. 1995). Parents were asked to rate how often they exhibit this behavior with their child on a 5‐point Likert scale ranging from Never (1) to Always (5). We selected items which capture reasoning, induction, and democratic participation to best capture parents’ encouragement of children's free and autonomous thought. For example, *“I help my child to understand the impact of their behavior by encouraging my child to think about the consequences of his/her actions.”* Reliability for this scale was acceptable (*α* = 0.70).

At the completion of the experiment both parent and child are debriefed and thanked for their participation and awarded a gift voucher.

## 7 Results

### 7.1 Preregistered Confirmatory Analyses

As with Study 1, we conducted six ANCOVAs (Table 5). In line with our preregistration, religiosity, sex, and diet category were included in the models as fixed factors, and parenting style (rather than political orientation) was included as a covariate. The omnibus ANCOVAs showed only a significant difference for speciesism. Tukey HSD follow up tests revealed that non‐vegetarians were significantly more speciesist than family vegetarians, *p* = 0.033, but independent vegetarians were not significantly different from non‐vegetarians, *p* = 0.093, or family vegetarians (*p* = 0.973). No differences were found for any other measures between the three dietary groups: CRT, identification of fearful faces, empathy/compassion, SDO, or disgust sensitivity.

**TABLE 5:** Standard deviations and means from the six ANCOVAs, comparing outcomes for the six metrics of personality and cognition across the three dietary groups.

					Family vegetarians		Independent vegetarians		Non‐vegetarians	
	*F*	*df*1	df2	*p*	Mean	SD	Mean	SD	Mean	SD
Cognitive reflection test (0–4)	0.29	2	223	0.747	1.11	1.13	1.08	0.98	0.93	1.03
Identifying fearful faces (0–4)	1.63	2	223	0.199	3.61	0.70	3.45	0.73	3.48	0.77
Disgust sensitivity (0–96)	0.60	2	223	0.551	54.94	12.95	54.50	12.88	53.98	11.99
Empathy/compassion (0–112)	1.81	2	223	0.166	61.67	14.39	62.56	14.69	58.35	14.39
SDO (1–7)	1.42	2	223	0.245	2.10	0.70	2.08	0.89	2.27	0.67
Speciesism (1–7)	3.65	2	223	**0.027**	1.82	0.90	1.85	0.80	2.29	1.05

*Note*: Bolding indicates a significant p value.

### 7.2 Preregistered Exploratory Analyses

We conducted a multinomial logistic regression identical to Study 1, including all six personality/cognitive scales, except that we included parenting style as a continuous predictor and did not include political orientation. The model provided a good fit for the data, *χ* ^2^ (18) = 36.14, *p* = 0.007. Pseudo *R* ^2^ values ranged from 0.051 (Cox & Snell) to 0.072 (McFadden). With all cognitive and personality measures, religiosity, parenting style and sex included in the model, we found that only religion significantly predicted diet category. Having religious parents was associated with a 3.31 times greater odds of being a non‐vegetarian compared to an independent vegetarian, (*B* = 1.20, 95% CI [0.45–1.94], *p* = 0.002), but not for being family compared to independent vegetarian (*B* = 0.29, 95% CI [−0.45 to 1.04], *p* = 0.445). No other predictors, including sex and parenting style, significantly predicted diet category.

In line with our pre‐registration, we report participants’ motivation for becoming vegetarian as a function of vegetarian type (family vs. independent; Table 6). The coding strategy was identical to Study 1. Two trained research assistants independently coded 100% of the responses for motivation to become vegetarian. Cronbach's alpha revealed that interrater reliability was good (*α* = 0.81). Again, the different motivation categories did not meet our pre‐registered criteria to perform chi‐square comparisons, with the exception of animal welfare. As such, we opted to compare the frequencies of vegetarians motivated by animal welfare (*N* = 75) and other reasons (*N* = 77) between independent and family vegetarians. Unlike our adult sample, we did not find that independent vegetarians were more likely to cite animal welfare motivations than family vegetarians, *p* = 0.146 (Fisher's exact *p* = 0.189).

**TABLE 6:** Children's motivation for diet across vegetarian groups.

	Family	Independent
Health	13	2
Environment	0	4
Social	27	4
Animal Welfare	39	36
Convenience	4	14
Other	0	2
Not reported	5	2
Total	88	64

Finally, like Study 1, we also investigated differences in some of the other motivation categories (social factors, health, and convenience) using Fisher's exact tests to account for the low *N* in many of the cells. We found that family vegetarians were significantly more likely to cite social factors as a motivation than independent vegetarians, *χ* ^2^(1, *N* = 152) = 13.62, *p* < 0.001 (Fisher's exact *p* < 0.001), OR = 6.64, 95% CI [2.19–20.12], as well as health as a motivation, *χ* ^2^(1, *N* = 152) = 5.65, *p* = 0.017 (Fisher's exact *p* = 0.025), OR = 5.37, 95% CI [1.17–24.72]. Conversely, independent vegetarians were more likely to cite convenience, *χ* ^2^(1, *N* = 152) = 10.66, *p* = 0.001 (Fisher's exact *p* = 0.002), OR = 0.17, 95% CI [0.05–0.55].

### 7.3 Exploratory Analyses

As in Study 1, we examined whether participants who were morally motivated differed from those who were not. To do this, we compared vegetarians who chose animal welfare as their primary motivator (moral vegetarians; *N* = 75) to all other participants (*N* = 161) and examined whether they varied on each of the six personality and cognitive measures. A series of Bonferroni‐corrected *t*‐tests (*α* = 0.008) revealed that, consistent with Study 1, moral vegetarians were lower in SDO and speciesism than other participants (Table 7). There were no differences between moral vegetarians and other participants for disgust sensitivity, identifying fearful faces, empathy/compassion, or cognitive reflection.

**TABLE 7:** Bonferroni corrected *t*‐tests for the six cognitive/personality scales, comparing morally motivated child vegetarians to all other participants.

					Other		Moral vegetarians		
	*t*	*p*	95% CI		Mean	SD	Mean	SD	Effect size
Cognitive reflection test	−0.02	0.985	−0.29	0.29	1.04	1.11	1.04	0.94	0.00
Identifying fearful faces	−1.13	0.262	−0.32	0.09	3.48	0.78	3.60	0.64	−0.16
Disgust sensitivity	1.67	0.097	−0.53	6.35	55.40	12.05	52.49	13.43	0.23
Empathy/compassion	−1.81	0.071	−7.64	0.32	59.57	14.58	63.23	14.17	−0.25
SDO	2.94	**0.004**	0.10	0.50	2.25	0.74	1.95	0.72	0.41
Speciesism	5.91	**<0.001**	0.42	0.83	2.20	1.02	1.57	0.60	0.75

*Note*: Levene's test for equality of variances was significant for speciesism, thus we report the statistics for equal variance not assumed and used a Welch's *t*‐test. Bolding indicates a significant p value.

## 8 Discussion

In Study 2 we examined differences in independent, family, and non‐vegetarian children across a range of personality and cognitive traits. We find no differences between dietary groups on any of our key measures, except that family vegetarians were lower than non‐vegetarians in speciesism, while independent vegetarians did not differ from either group. This again did not persist in a model with all traits and demographics included. Relatedly, and unexpectedly, independent vegetarians were less likely to be religious than non‐vegetarians. Thus, religiosity appears to be a stronger predictor of dietary status than speciesist views in our child sample.

We also compare dietary motivations. Unlike Study 1, independent vegetarians were not significantly more likely to cite animal welfare as a motivator than family vegetarians. This could suggest that the independent vegetarians in Study 1 (adults) may have retrospectively attributed moral motivations to their decision to become vegetarian, even if they had other reasons as children. Reflecting Study 1, independent vegetarians were again more likely to cite convenience and less likely to cite social factors. Unexpectedly, and different from, Study 1, they were less likely to cite health than family vegetarians.

Finally, in an exploratory analysis, we again examined the differences between morally motivated vegetarians and all other participants, revealing that morally motivated vegetarians were lower in speciesism and SDO than other participants.

## 9 General Discussion

A growing body of research has aimed to characterize extraordinary altruists—people who engage in costly altruistic acts to benefit distant others (Amormino et al. 2022; Brethel‐Haurwitz et al. 2018; MacFarquhar 2015; Marsh et al. 2014; Vekaria et al. 2017). To date, this research has mostly focused on adults. We examine one possible expression of extraordinarily altruistic motivation in children: the decision to stop eating meat while being raised in a meat‐eating family (i.e., independent vegetarianism). Employing a retrospective adult sample (adults who adhered to the relevant diet category during childhood; Study 1) and a developmental sample (8–11‐year‐olds; Study 2) of American participants, we investigated what might differentiate independently vegetarian children from family vegetarian and non‐vegetarian children. We examined whether these three groups differed on six cognitive and personality measures: empathy/compassion, disgust sensitivity, cognitive reflection, fearful facial recognition, SDO, and speciesism. We found a number of measures that differentiated vegetarians from non‐vegetarians. However, against our key prediction, we did not identify any differences between family and independent vegetarians. We explore possible explanations for this.

It may be that vegetarians, both family and independent, share a set of traits that predispose them to this diet—such as greater concern for animals, high empathy, and low hierarchical worldviews, as we identify here. Under this view, these children may have ultimately become vegetarian regardless of whether they were raised by other vegetarians or by omnivores. That is, the role of family environment might be minimal.

Contrasting this, it may be that we missed a particular source of social influence. We assumed that independent vegetarians acted outside of the norm when deciding to become vegetarian because it was not modeled by their immediate family. However, it is also possible it was modelled by extended family members, peers, or on social media—all of which are particularly influential to young people (Tolbert and Drogos 2019; Wallis and Loy 2021). Future work could map social networks more thoroughly to better understand the role of social influence.

Another interpretation is that the relationship between our diet and values is bidirectional. That is, while many people become vegetarian because they have certain values (e.g., they care about animals), being a vegetarian might also change one's values (e.g., increase the amount that they care for animals). Under this view, becoming vegetarian for any reason might change your values and beliefs, which could explain why we found few differences between independent and family vegetarians in either sample. It also aligns with the self‐reported dietary motivation: independent and family vegetarians cited animal welfare at similar rates in the child sample, while independent vegetarians were more likely than family vegetarians to cite animal welfare in the adult sample. That is, people may become more motivated by animal welfare and less motivated by other reasons over time. This perspective is supported by both dissonance‐based accounts (the Meat Paradox; Bastian et al. 2012; Gradidge et al. 2021; Loughnan et al. 2010), as well inference‐based accounts (Self‐perception Theory; Bem 1972). While we cannot distinguish the specific mechanisms with our data, the bidirectional account seems plausible and is an important topic for future research.

While we found no differences between vegetarian groups, we did find that both vegetarian groups (family and independent) differed from non‐vegetarians on a range of measures, though these results were predominantly contained to the adult sample. First, we find robust evidence that childhood vegetarians are lower in speciesism than non‐vegetarians, reflecting findings in the existing literature with adult (Dhont and Ioannidou 2024; Leach et al. 2022). However, it is worth noting that, in the developmental sample, only family vegetarians were lower in speciesism than non‐vegetarians, while independent vegetarians did not reach the threshold for significance. It is unclear if this an issue of power or whether other demographic factors (e.g., religiosity) simply play a more prominent role.

We also found that childhood vegetarians scored higher on empathy/compassion. These findings support links between empathy and altruistic motivation, for which evidence is currently ambivalent (Amormino et al. 2022; Jordan et al. 2016; Rhoads et al. 2023; Wilks et al. 2024). In this sample, we also find evidence that vegetarians are higher in disgust sensitivity and lower in SDO. However, this pattern only emerged when we excluded participants who were raised eating meat but were vegetarian at the time of completing the survey (i.e., they were classified as meat eaters, despite currently having a vegetarian diet), and did not reliably persist when other traits were included in our models. As such, while the relationship is there, other factors may be stronger predictors. Nonetheless, our findings mirror the existing literature suggesting high disgust sensitivity and low SDO among meat avoiders (Becker et al. 2022; Becker and Lawrence 2021; Rosenfeld 2018), as well as extending work that has previously linked speciesism to other prejudices, such as SDO (Caviola et al. 2019; Dhont et al. 2016). Interestingly, it also contrasts with a body of work linking pathogen avoidance to higher social dominance orientation and other measures of conservativeness (Terrizzi et al. 2013).

We also note a number of traits in which we find no differences between groups. This reflects similarities between independent, family, and non‐vegetarian children in past literature. For example, Hussar and Harris (2010) find that all three groups think about meat eating as a personal choice, while Hussar and Harris (2018) find that all three diet groups judge harm to humans and animals in similar ways. Here, we found no differences in the ability to identify fearful faces. This contrasts with existing literature, which has found that the ability to identify fearful facial expressions is higher in non‐directed kidney donors (Marsh et al. 2014) and charitable pledgers (Wilks et al. 2024). Specific task differences may explain this result (i.e., Marsh and colleagues used a different task, while Wilks and colleagues used the same task with additional facial expressions). Indeed, participants scored quite highly overall and internal consistency was low, suggesting that the task may have been too easy. However, another potential explanation for this null is that the connection between recognizing certain emotions and altruistic motivation is target specific; the ability to better acknowledge the fear or suffering of non‐human animals would be a more predictive indicator of altruistic motivation and behavior toward animals. It would be good to replicate this with an improved measure before strong conclusions are drawn.

We also found no group‐level differences on measures of cognitive reflection. This contrasts past work finding that *Giving What We Can* pledgers are higher in related traits such as actively open‐minded thinking and need for cognition (Wilks et al. 2024). Importantly, *Giving What We Can* focuses on *effective giving*, thus it is plausible that these pledgers would score higher on measures related to reasoning and logic than other groups of extraordinary altruists. Finally, in Study 2 we found no evidence that parenting style was related to children's dietary choices. This contrasts with prior work showing that adults raised by egalitarian parents with non‐authoritarian parenting styles were more likely to engage in extraordinary altruism (Oliner and Oliner 1988). Again, in both of these cases, we recommend further research explores the nuances of these relationships.

Our investigation is premised on the idea that there is a moral motivation underpinning independent vegetarianism. As such, we also compared vegetarians who cited moral reasons (i.e., animal welfare) as the primary motivator for their diet to all other participants. Across both studies, morally motivated vegetarians were lower in SDO and speciesism and, in the adult sample, higher in empathy/compassion. This is similar to the previously discussed findings, though it suggests a more meaningful role of SDO. Notably, other work has linked vegan diets to lower rates of SDO and similar traits (e.g., right wing authoritarianism; Hodson and Earle 2018), suggesting that this nonhierarchical worldview may be particularly salient for vegetarians who are morally motivated.

Finally, some methodological limitations should be acknowledged in addition to those discussed earlier. First, we worked with a sample of American participants. Research with adults has shown that different values underpin vegetarian motivations across countries (e.g., Ruby et al. 2013), thus the pattern of results identified here may not persist in other cultural contexts. We also adapted validated adult scales for children, which may partially explain the largely null results in Study 2. Employing scales developed for children would be an important next step, such as the child CRT‐D scale (Young and Shtulman 2020) and the child SDO‐6 scale (Cadamuro et al. 2022)—both of which were published after our study was preregistered and data collection had begun.

We set out to understand extraordinary altruistic motivation in childhood, by examining how independent vegetarians differed from family and non‐vegetarians. We did not capture any traits that set apart independent vegetarians from family vegetarians, suggesting that the choice to become vegetarian independently may not be a helpful proxy for extraordinarily altruistic motivation. However, we did identify a number of factors that separate vegetarians from meat eaters in childhood, namely speciesism, SDO, and empathy and compassion—though these results were primarily found in the retrospective adult sample. This adds new data to the small body of literature explicitly examining independent vegetarianism in childhood (Hussar and Harris 2010, 2018), identifying traits that characterize both independent and family vegetarians.

## Author Contributions

**Matti Wilks**: conceptualization, writing – original draft, methodology, writing – review and editing, formal analysis, data curation, project administration, investigation. **Alexa Sacchi**: methodology, project administration, data curation, writing – review and editing. **Beatrice Turner**: writing – review and editing, formal analysis, project administration, data curation. **Paul Bloom**: funding acquisition, writing – review and editing, supervision, methodology, conceptualization.

## Conflicts of Interest

No COI to declare.

## Data Availability

The data that support the findings of this study are openly available in Open Science Framework at https://osf.io/xb2s9/.

## Endnotes

1 We specified that the responses to our inquiry would be anonymous, thus we do not share the names of contributors here.

2 We initially set the criteria to 6 months, but reduced it due to difficulty with recruitment.
